# Invasive Listeriosis in an Older Adult With a History of Raw Crab Consumption: A Case Report

**DOI:** 10.7759/cureus.116533

**Published:** 2026-09-19

**Authors:** David Fernando Narvaez-De Los Rios, Daniel Ibanez Vasquez, Valentina Erazo-Fernández, Chimezirim Ezeano, Jairo A. Eraso-Zamora

**Affiliations:** 1 Cardiology, Centro de Investigaciones Clínicas, Fundación Valle del Lili, Cali, COL; 2 Internal Medicine, Universidad Privada del Valle, Cochabamba, BOL; 3 Faculty of Health Sciences, Universidad ICESI, Cali, COL; 4 Department of Internal Medicine, Aurora Health Care, Aurora Sinai Medical Center and Aurora St. Luke’s Medical Center, Milwaukee, USA; 5 Department of Infectious Diseases, Aurora Health Care, Aurora Sinai Medical Center and Aurora St. Luke’s Medical Center, Milwaukee, USA

**Keywords:** bacteremia, case report, invasive listeriosis, listeria monocytogenes, raw crab

## Abstract

*Listeria monocytogenes* can cause severe invasive infection in older and medically vulnerable patients, and pinpointing a potential exposure may require a detailed dietary history. We report the case of an 84-year-old woman with end-stage renal disease on hemodialysis, type 2 diabetes mellitus, cardiovascular disease, and vascular dementia who presented with chest pain and mild dyspnea and then developed fever, hypotension, tachycardia, and a single episode of diarrhea, which prompted evaluation for sepsis. Blood cultures turned positive in both aerobic and anaerobic bottles after approximately 22 hours of incubation and were subsequently identified as *Listeria monocytogenes*. Empiric vancomycin was stopped, and targeted therapy with intravenous ampicillin and adjunctive gentamicin was started. Repeat blood cultures remained negative during Infectious Diseases follow-up. There were no local signs of infection at her left upper-extremity arteriovenous fistula, transthoracic echocardiography showed no reported valvular vegetations, and no clinical evidence of central nervous system involvement was found. Collateral dietary history obtained during hospitalization revealed that she had eaten raw crab before admission. Because the interval between consumption and symptom onset could not be established and the implicated food was unavailable for microbiological testing, the exposure was considered plausible but unconfirmed. The patient improved clinically and was discharged in stable condition. This case highlights the importance of considering invasive listeriosis in older, medically vulnerable patients with bacteremia or sepsis, evaluating for possible invasive complications, and obtaining detailed collateral dietary histories, while avoiding unsupported attribution of causality to a suspected food exposure.

## Introduction

*Listeria monocytogenes* is a Gram-positive, non-spore-forming, motile, facultatively anaerobic bacterium that is widely distributed in environmental, animal, and food reservoirs. Its ability to grow at refrigeration temperatures and to persist in food-processing environments facilitates contamination of refrigerated and ready-to-eat products [1,2]. Foodborne transmission is the primary route of human infection, with dairy products, processed meats, raw foods, and fresh produce among the most frequently recognized vehicles [1]. Although seafood is implicated less often, *Listeria monocytogenes* has been detected in raw crab and crab-processing environments, and crabmeat products have previously been linked to listeriosis outbreaks [3-5].

In immunocompetent individuals, infection is generally asymptomatic or limited to a self-limited febrile gastroenteritis. In contrast, older adults, pregnant women, neonates, and individuals with impaired cell-mediated immunity are at increased risk of invasive listeriosis, which typically presents as bacteremia or central nervous system infection and is associated with substantial mortality [1,6]. In United States surveillance data from 2009 to 2011, 58% of invasive cases occurred among adults aged 65 years or older; incidence in this age group was 4.4 times that of the overall population, and the case-fatality proportion was 24% [7].

The published literature on crab has primarily documented *Listeria monocytogenes* contamination in raw crab and crab-processing environments, as well as outbreaks associated with crabmeat products [3-5]. This report describes invasive listeriosis presenting as bacteremia in an older adult with multiple predisposing conditions, for whom collateral dietary history revealed raw crab consumption before hospitalization. Because the interval between consumption and symptom onset could not be established and the implicated food was unavailable for microbiological testing, the exposure is considered plausible but unconfirmed. The contribution of this case lies in illustrating an initially atypical presentation of invasive listeriosis in a highly susceptible patient and in demonstrating the diagnostic value of obtaining a detailed collateral dietary history when evaluating bacteremia or sepsis without an apparent source.

## Case presentation

An 84-year-old Korean woman with a medical history of end-stage renal disease requiring hemodialysis, type 2 diabetes mellitus, hypertension, coronary artery disease, congestive heart failure, peripheral vascular disease, chronic anemia, and vascular dementia presented to the emergency department on June 1, 2026, with chest pain and mild dyspnea. On arrival, she was hemodynamically stable. Physical examination revealed scattered pulmonary crackles, without other clinically significant findings. Initial laboratory investigations showed a white blood cell count of 7.8 × 10³/µL, anemia, hypoalbuminemia, and impaired renal function consistent with her underlying chronic conditions. High-sensitivity cardiac troponin I was elevated at 1,596 ng/L on admission and decreased to 1,254 ng/L on repeat testing (reference range: < 52 ng/L). Given her chest pain, extensive cardiovascular history, and elevated troponin, Cardiology evaluated the patient.

The available clinical documentation characterized the finding as a “troponin leak” and did not establish a definitive diagnosis of acute coronary syndrome. Conservative medical management was recommended, and she remained on telemetry. Serial laboratory findings are summarized in Table 1. Chest radiography demonstrated vascular congestion, bilateral pleural effusions greater on the right, and bibasilar opacities that were grossly similar to prior imaging, without a new focal consolidation to suggest pneumonia as the source of infection (Figure 1). Nephrology was consulted to continue her scheduled hemodialysis.

**Table 1:** Serial laboratory findings during hospitalization BUN: blood urea nitrogen; WBC: white blood cell count

Laboratory parameter	Reference range	Admission	Day 1	Day 2	Day 3	Day 4
WBC (×10³/µL)	4.2–11.0	7.8	—	6.6	7.1	7.6
Hemoglobin (g/dL)	12.0–15.5	9.9	—	8.9	9.4	9.3
Hematocrit (%)	36.0–46.5	30.7	—	27.8	28.6	29.2
Platelets (×10³/µL)	150–450	149	—	144	147	155
BUN (mg/dL)	6–20	53	22	28	—	24
Creatinine (mg/dL)	0.51–0.95	5.53	3.02	3.77	5.02	3.12
Procalcitonin (ng/mL)	—	—	4.73	—	—	—
High-sensitivity troponin I (ng/L)	< 52	1,596	1,254	—	—	—

**Figure 1:**
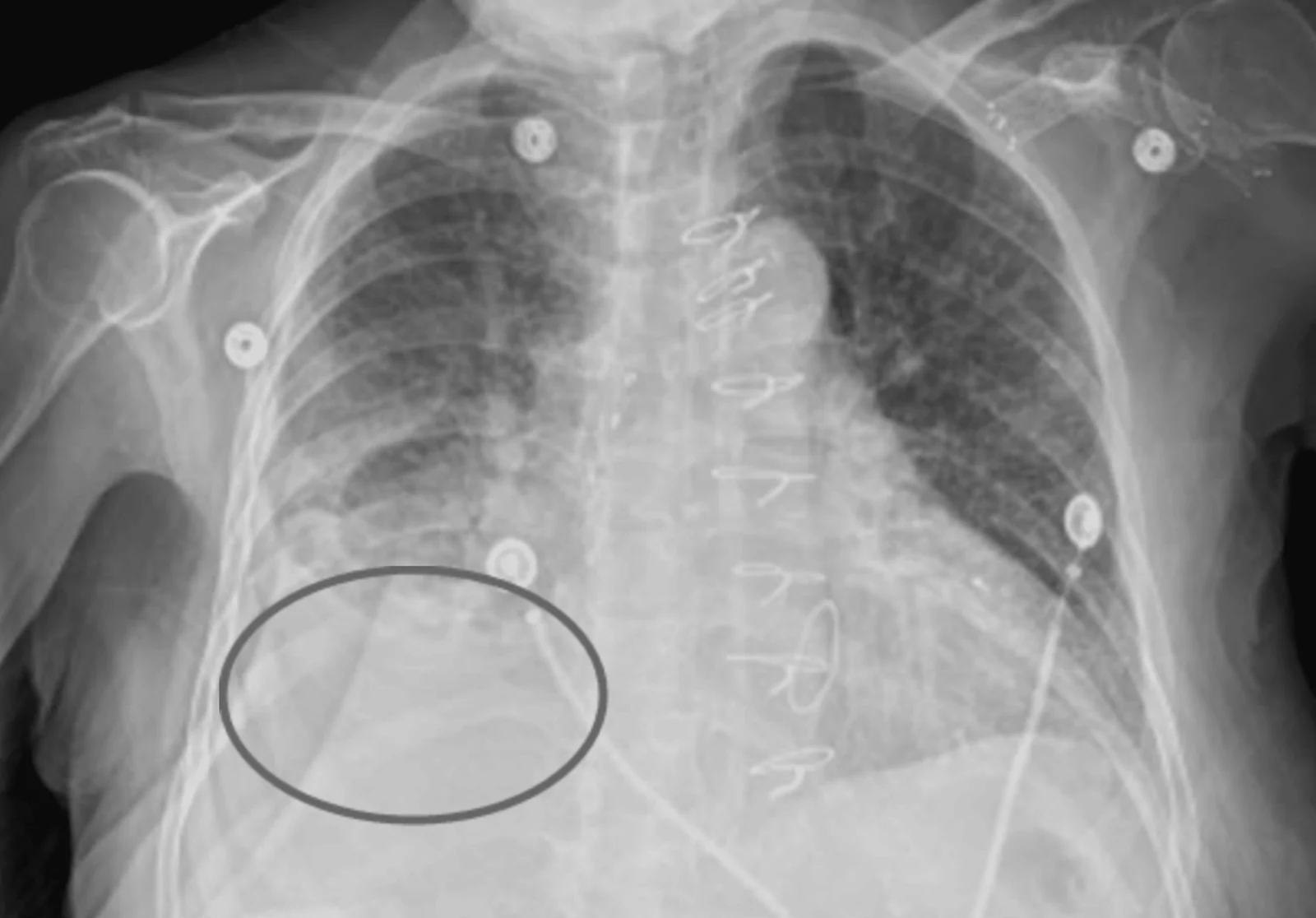
Chest radiograph obtained at presentation Anteroposterior chest radiograph demonstrating vascular congestion, bilateral pleural effusions greater on the right, and bibasilar opacities similar to prior imaging. No new focal air-space consolidation was identified to suggest pneumonia as the source of infection

After admission, the patient experienced a single episode of diarrhea and subsequently developed fever, hypotension, and tachycardia in the setting of atrial fibrillation, prompting evaluation for sepsis without an initially identifiable source. Blood cultures were obtained, and empiric antimicrobial therapy with vancomycin was initiated with dosing guided by the pharmacy service. Her procalcitonin level was elevated at 4.73 ng/mL, and a respiratory pathogen panel was negative. After approximately 22 hours of incubation, both aerobic and anaerobic blood-culture bottles became positive for Gram-positive bacilli, which were subsequently identified as *Listeria monocytogenes*. 

Following identification of *Listeria monocytogenes*, empiric vancomycin was discontinued and targeted therapy with intravenous ampicillin 2 g every 12 hours and adjunctive gentamicin was initiated. Additional blood cultures were obtained and remained negative during Infectious Diseases follow-up. Antimicrobial susceptibility testing demonstrated susceptibility to ampicillin and penicillin G, while trimethoprim-sulfamethoxazole was reported as nonsusceptible (Table 2). She continued hemodialysis through a left upper-extremity arteriovenous fistula, which showed no local signs of infection.

**Table 2:** Antimicrobial susceptibility profile of the blood isolate Organism identification and antimicrobial susceptibility testing were performed by the clinical microbiology laboratory. Interpretive categories are reported as provided by the laboratory MIC: minimum inhibitory concentration

Antimicrobial agent	Interpretation	MIC (µg/mL)
Ampicillin	Susceptible	0.12
Penicillin G	Susceptible	0.12
Trimethoprim-sulfamethoxazole	Nonsusceptible	1.0

Given the presence of *Listeria monocytogenes* bacteremia, transthoracic echocardiography was performed as part of the evaluation for infective endocarditis and showed no reported valvular vegetations. Neurologic examinations remained nonfocal and consistent with her baseline status, and head CT showed no acute intracranial abnormalities. No clinical evidence of central nervous system involvement was identified during hospitalization.

By hospital day four, the patient had clinically improved. She was afebrile and normotensive, awake and in no acute distress, and reported no fever, chills, nausea, abdominal pain, or further diarrhea. Additional dietary history obtained during hospitalization revealed raw crab consumption before admission. The precise date of consumption and the interval between this exposure and symptom onset could not be established. In the absence of another microbiologically confirmed source, raw crab consumption was considered a plausible exposure. However, because the implicated food was unavailable for microbiological testing, a definitive causal association could not be established.

The patient completed a two-week course of intravenous ampicillin 2 g every 12 hours and a one-week course of gentamicin, with dosing guided by the pharmacy service. She remained hospitalized for continued management and monitoring until the acute infection and her underlying comorbidities were clinically stable, and was subsequently discharged in stable condition with outpatient follow-up.

## Discussion

Invasive listeriosis occurs predominantly in susceptible groups, particularly older adults and patients with impaired cell-mediated immunity or substantial comorbidities [1,6,7]. Advanced age, diabetes mellitus, and renal failure are recognized risk factors for invasive disease [1,6,7]. Our patient had several of these predisposing conditions, including advanced age (84 years), end-stage renal disease on hemodialysis, and type 2 diabetes mellitus. Her initial presentation with chest pain and mild dyspnea was not suggestive of foodborne infection. Fever, hypotension, and tachycardia developed during hospitalization, which prompted evaluation for sepsis without an apparent source. The single episode of diarrhea was compatible with gastrointestinal exposure but nonspecific. After ingestion, *Listeria monocytogenes* may cross the intestinal barrier and disseminate hematogenously, and progression to invasive disease is influenced by both bacterial and host factors [6].

Evaluation of *Listeria monocytogenes* bacteremia should consider potential invasive complications and alternative infectious foci. In this patient, serial neurologic examinations remained nonfocal and consistent with her baseline status, and head computed tomography showed no acute intracranial abnormality. Transthoracic echocardiography showed no reported valvular vegetations. Her left upper-extremity arteriovenous fistula also showed no local evidence of infection, and chest imaging did not show a new focal consolidation suggestive of pneumonia (Figure 1). Additional blood cultures remained negative during Infectious Diseases follow-up. Together with her subsequent clinical improvement, these findings supported managing the infection as *Listeria monocytogenes* bacteremia without an identified deep-seated infectious focus.

Penicillin and aminopenicillins, including ampicillin, are considered first-line antimicrobial therapy for invasive *Listeria monocytogenes* infection, although controlled trials defining the optimal regimen and duration are lacking [6]. In this patient, empiric vancomycin was stopped once the organism was identified as *Listeria monocytogenes*, and treatment was narrowed to intravenous ampicillin with adjunctive gentamicin. Gentamicin has been used as adjunctive therapy because of its potential synergistic activity with beta-lactams, although the clinical benefit of combination therapy remains uncertain and its toxicity warrants particular consideration in patients with renal dysfunction [6]. Because she had end-stage renal disease on hemodialysis, antimicrobial dosing was adjusted with pharmacy guidance. She completed a two-week course of intravenous ampicillin and a one-week course of gentamicin. Management of her bacteremia took into account the absence of evidence of endocarditis or clinically apparent central nervous system involvement, negative subsequent blood cultures, and sustained clinical improvement.

*Listeria monocytogenes* is widely distributed in environmental and food reservoirs, and seafood may become contaminated during harvesting, processing, transportation, storage, handling, or preparation [1,2]. Evidence on crab supports the biological plausibility of the exposure identified in this case. Across seven blue crab processing plants, *Listeria monocytogenes* was recovered from 4.5% of raw crabs, 0.2% of cooked crabmeat samples, and 2.1% of environmental samples [4]. Whole-genome sequencing has identified persistent strains in crabmeat-processing environments associated with two outbreaks of listeriosis [3], and a small outbreak was potentially linked to imitation crabmeat, in which the clinical and food isolates were molecularly indistinguishable [5]. Reports of other unusual raw animal-food exposures further illustrate the importance of obtaining detailed dietary histories in patients with invasive listeriosis [8].

In our patient, collateral dietary history revealed that she had eaten raw crab before hospitalization. However, the implicated food was unavailable for microbiological testing, the interval between consumption and symptom onset could not be established, and no traceback investigation was performed. Raw crab should therefore be considered a plausible epidemiologic exposure rather than an established source of infection. Likewise, the point of potential contamination, whether during harvesting, processing, storage, handling, or preparation, cannot be determined.

The broader clinical relevance of the dietary history therefore lies not in the attribution of infection to a particular food but in the value of obtaining individualized exposure information when evaluating bacteremia or sepsis without an apparent source. The patient’s Korean background provides demographic context but should not be interpreted as an independent risk factor for listeriosis. Dietary practices vary within and across populations, and clinicians should ask nonjudgmental questions about raw or undercooked foods, preparation methods, and storage practices rather than making assumptions based on ethnicity. When cognitive impairment limits direct history-taking, collateral information from relatives or caregivers may be particularly valuable, as this case demonstrates. Food-safety counseling should likewise be tailored to host susceptibility and documented dietary practices [2,7,9].

A strength of this case report is the microbiological confirmation of invasive *Listeria monocytogenes* infection, together with a detailed evaluation of a clinically vulnerable patient whose initial presentation was atypical. Important limitations include the absence of microbiological testing of the suspected food exposure, the unknown exposure-to-symptom interval, and the possibility of recall bias. Therefore, this case does not establish raw crab as the source of infection. Its contribution lies in illustrating invasive listeriosis in a highly susceptible older adult, emphasizing the importance of evaluating for potential invasive complications, and demonstrating how a detailed collateral dietary history can identify potentially relevant exposures when the source of bacteremia is initially unclear.

## Conclusions

Invasive *Listeria monocytogenes* infection should be considered in older and medically vulnerable patients presenting with bacteremia or sepsis, even when the initial presentation is atypical and gastrointestinal manifestations are minimal. In this case, collateral dietary history identified raw crab consumption before hospitalization; however, the source of infection could not be established because the interval between exposure and symptom onset was unknown and the implicated food was unavailable for microbiological testing. This case report highlights the importance of recognizing invasive listeriosis in susceptible hosts, evaluating for potential invasive complications, providing appropriate directed antimicrobial therapy, and obtaining detailed collateral exposure histories when the source of bacteremia is initially unclear.
